# Occupational Violence against Brazilian Nurses

**Published:** 2018-11

**Authors:** Alessandro Leite CAVALCANTI, Eduardo dos Reis BELO, Emanuella de Castro MARCOLINO, Américo FERNANDES, Yuri Wanderley CAVALCANTI, Danielle Franklin de CARVALHO, Ana Maria Gondim VALENÇA, Alidianne Fabia Cabral CAVALCANTI, Wilton Wilney Nascimento PADILHA

**Affiliations:** 1. Post-Graduate Program in Public Health, State University of Paraiba, Campina Grande, PB, Brazil; 2. Dept. of Clinic and Social Dentistry, Federal University of Paraiba, Joao Pessoa, PB, Brazil; 3. School of Dentistry, State University of Paraiba, Campina Grande, PB, Brazil

**Keywords:** Workplace violence, Occupational risks, Occupational health nursing

## Abstract

**Background:**

We evaluated the prevalence and risk factors of workplace violence against Brazilian nurses in 2014.

**Methods:**

The study’s population comprised of 112 nurses working in teams of Family Primary Care Units and Primary Care Health Centers. Those nurses were asked to answer a questionnaire that addressed the socio-demographic information, the professional routine and the occupational violence faced (types, frequency and characteristics of perpetrators). Data were analyzed using the SPSS software.

**Results:**

Most of nurses were female (94.6%), aged between 34–43 yr (38.4%), living with a partner (60.7%) and having a weekly workload of 40 h (90.1 %). The prevalence of violence was 73.2%. Predominantly, occupational violence comprised of verbal violence (67.0%) and psychological harassment (bullying −27.1%). Patients (81.1%) and caregivers (83.1%) were responsible for verbal violence, whilst the heads of teams (78.3%) and other health professionals (41.7 %) practiced bullying. The risk factors more frequently reported were the lack of safety in the workplace (73.2%) and the aggressive behavior of patients (67%). The occupational violence was not statistically associated with the gender, professional experience, experience at primary health care, weekly working hours, or working shift. The type of violence faced was not either statistically associated with gender, marital status, professional experience, weekly working hours, or working shift.

**Conclusion:**

Occupational violence has high prevalence among Brazilian nurses working at primary health care system. Verbal violence is more prevalent and frequently practiced by patients. The lack of safety in the workplace is the main risk factor associated with occupational violence faced by nurses.

## Introduction

Violence in the working place is a global public health problem (1–4). It is considerate to be a reflection of the level and wave of violence suffered in the whole society (5). The National Institute for Occupational Safety and Health (NIOSH) defines workplace violence as “violent acts, including physical assaults and threats of assault, directed toward persons at work or on duty” (6).

Workplace violence takes many forms, such as verbal abuse, aggression, harassment, bullying, physical violence, and it may include various types of perpetration (7). There is a consensus that the most commonly encountered violence is verbal abuse (8–10).

The reported frequency of violence is increasing in the health care sector (11). Workplace violence directed at nurses has become an occupational health problem once nurses are considered the health care workers most likely at risk, and female nurses are considered at the greatest risk (12).

Numerous studies on occupational violence have been conducted in many countries (1,8,13,14). The prevalence of violence in the nursing staff ranged from 38.9% in Thailand (15) to 76% in Hong Kong (9).

Violence against Brazilian nurses has not been studied previously. The aim of this study was to determine the prevalence and risk factors of workplace violence against Brazilian nurses.

## Materials and Methods

This cross-sectional study was conducted between Mar and May 2014, in the city of Campina Grande, Northeast, Brazil. This city has approximately 385213 inhabitants and it is territorially divided into 6 health-sanitary districts, under management of the local administration of the public health system. Those districts include 100 Family Primary Care Units and three Primary Care Health Centers, where 112 nurses are employed.

Data was collected using a questionnaire previously validated (16). This questionnaire included socio-demographic information (gender, age and marital status) and the professional characterization and routine (graduation, professional experience, experience at primary care system, weekly working hours, working shift). Additionally, using the same questionnaire, nurses were asked to report their workplace violence experience during the past year. The occupational violence was assessed considering: I) the frequency; II) the type of violence (physical abuse, verbal abuse, psychological harassment and/or sexual harassment); III) the perpetrator characteristics (position and gender); IV) the working shift that aggression occurred; and V) possible risk factors.

Data analysis involved descriptive statistics (frequency distribution) and analytic statistics. To test the association between the occurrence of violence and demographic and professional variables a process of bivariate analysis was conducted, using the exact versions of the nonparametric Pearson’s chi-squared test or Fisher’s exact test. The level of statistical significance was set at 5% with a confidence interval of 95%.

This study followed all the ethical guidelines recommended by the international scientific community and by the Brazilian legislation. The Ethics Committee of Human Research from the State University of Paraiba has previously approved the study. All participants/guardians agreed and signed an informed consent form.

## Results

Most nurses were female (94.6%), aged between 34–43 yr (38.4%), living with a partner (60.7%). With regards to the professional experience, most of them were specialists (88.4%), working as a nurse for 11 to 15 yr (23.2%), and have been working at the primary care for 6 to 10 yr (36.6%). Nurses have, predominantly, 40 h weekly workload (90.1%), working full time during the week (93.7%) (Table 1).

**Table 1:** Distribution of nurses according to socio-demographic variables and professional experience (n=112). Campina Grande, Brazil, 2014

***Variable***	***Frequency***	
	***n***	***%***
**Gender**		
Male	6	5.4
Female	106	94.6
**Age(yr)**		
23 - 33	33	29.5
34 - 43	43	38.4
44 - 53	29	25.9
54 - 63	7	6.2
**Marital Status**		
With a partner	68	60.7
Single	44	39.3
**Professional education**		
Graduate	1	0.9
Specialist	99	88.4
Master of Science in Nursing (MSN)	12	10.7
**Length of professional experience (years)**		
1 to 5	19	17
6 to 10	25	22.3
11 to 15	28	25
16 to 20	18	16.1
21 to 26	11	9.8
>27	11	9.8
**Experience at primary health care system (years)**		
< 1	20	17.9
1 to 5	34	30.3
6 to 10	41	36.6
11 to 20	14	12.5
>20	3	2.7
**Weekly working hours**		
20	7	6.3
32	3	2.7
36	1	0.9
40	101	90.1
**Working shift**		
Half time	7	6.3
Full time	105	93.7

Considering the occupational violence faced, 73.2% reported some type of violence within the workplace. The 12-month prevalence of verbal abuse (67%) was the highest, followed by psychological harassment (27.1%), sexual violence (4.2%), and physical violence (1.7%).

With regards to the frequency that occupational violence occurred, nurses reported four or more events of verbal violence (30%), whilst psychological harassment was reported only once (39%). Perpetrators of verbal violence were predominantly patients (81.1%) and their accompanying person (83.1%). However, heads of teams (78.3%) and other health professionals (41.7%) were the main perpetrators of psychological harassment. Violence was practiced by individuals of both genders, with no differences between morning or afternoon periods (Table 2).

**Table 2:** Distribution of nurses according to variables frequency of violence, perpetrator, gender of perpetrator and time that violence occurred, Campina Grande, Brazil, 2014

***Variables***		***Type of Violence***							
		***Physical***		***Verbal***		***Psychological***		***Sexual***	
		***n***	***%***	***n***	***%***	***n***	***%***	***n***	***%***
Frequency	Once	2	4.9	19	46.3	16	39.0	4	9.8
	Twice	-	-	15	78.9	3	15.8	1	5.3
	Three times	-	-	15	88.2	2	11.8	-	-
	Four times or more	-	-	30	73.2	11	26.8	-	-
Perpetrator	Patient	2	2.7	60	81.1	8	10.8	4	5.4
	Accompanying person	-	-	49	83.1	10	16.9		
	Health professional^1^	-	-	20	55.5	15	41.7	1	2.8
	Head of team	-	-	5	21.7	18	78.3	-	-
	Others^2^	-	-	3	75.0	1	25.0	-	-
Gender of the perpetrator	Male	-	-	8	53.3	4	26.7	3	20.0
	Female	2	3.2	42	67.7	18	29.1	-	-
	Male and female	-	-	29	70.7	10	24.4	2	4.9
Time of aggression	Morning	1	2.0	32	64.0	14	28.0	3	6.0
	Afternoon	1	5.6	12	66.6	4	22.2	1	5.6
	Both	-	-	35	70.0	14	28.0	1	2.0

1 Another primary care team members, such as medical doctors, dentists, auxiliary nurse, communitarian health agent, among others.

2 Others perpetrators refer to administrative people and visitors.

The risk factors more frequently reported by nurses were the lack of security within the workplace (73.2%) and violent behavior of patients (67%) (Table 3). Statistically, significant association was not verified between occupational violence and the variables gender, professional experience, experience at primary health care, weekly working hours, or working shift (Table 4). Similarly, bivariate analysis revealed that the type of violence faced was not statistically associated with variables gender, marital status, professional experience, weekly working hours, or working shift (Table 5).

**Table 3:** Distribution of risk factors to occurrence of violence according to the opinion of primary care nurses, Campina Grande, Brazil, 2014

***Risk Factors to occurrence of violence***	***Frequency***	
	***n***	***%***
Lack of security in the workplace	82	73.2
Violent behavior of patients	75	67.0
Violent behavior of accompanying person	69	61.6
Poor team training	66	58.9
Poor and/or inadequate structure	46	41.1
Understaffing	35	31.3
Deficient service provided to patients	27	24.1
Long queues waiting for service	26	23.2
Other reasons^1^	29	25.9

1 Other reasons refer to poor trained nursery team; violent behavior of the head of team; use of drugs; lack of services; inefficient health care system; administrative problems; lack of knowledge about rights and duties of the patients; Shortage of medicines and supplies; delay in examination marking and delayed time professionals

**Table 4:** Distribution of the prevalence of occupational violence according to gender; professional experience (in years); experience at Primary Health Care (in years); weekly working hours; and working shift. Campina Grande, Brazil, 2014

***Variable***		***Faced some type of violence***				***P-value***	***PR***
		***Yes***		***No***			
		***n***	***%***	***n***	***%***		
Gender	Male	5	83.3	1	16.7	>0.05	1.88
	Female	77	72.6	29	27.4		(0.21–16.81)
Professional Experience (years)							
	1 to 15	50	69.4	22	30.6	>0.05	0.56
	16 or more	32	80	8	20		(0.22–1.43)
Experience at Primary Health Care (years)							
	1 to 5	38	70.4	16	29.6	>0.05	0.75
	6 or more	44	75.9	14	24.1		(0.32–1.74)
Weekly working hours							
	< 40 h	7	63.6	4	36.4	>0.05	0.6
	40 h	75	74.3	26	25.7		(0.16–2.24)
Working shift							
	Half time	3	42.9	4	57.1	>0.05	0.24
	Full time	79	75.2	26	24.8		(0.05–1.17)

PR = Prevalence Ratio

**Table 5:** Frequency distribution of the type of violence according to gender; marital status, professional experience (in years); weekly working hours; and working shift. Campina Grande, Brazil, 2014

***Variables***	***Type of violence***																			
	***Physical***				***P-value***	***Verbal***				***P-value***	***Psychological***				***P-value***	***Sexual***				***P-value***
	***Yes***		***No***			***Yes***		***No***			***Yes***		***No***			***Yes***		***No***		
	***n***	***%***	***n***	***%***		***n***	***%***	***n***	***%***		***n***	***%***	***n***	***%***		***n***	***%***	***n***	***%***	
**Gender**																				
Male	-	-	6	100.0	>0.05	5	83.3	1	16.7	>0.05	3	50.0	3	50.0	>0.05	1	17.0	5	83.3	>0.05
Female	2	1.9	104	98.1		74	69.8	32	30.2		29	27.4	77	72.6		4	3.8	102	96.2	
**Marital Status**																				
Single	2	4.5	42	95.5	>0.05	31	70.5	13	29.5	>0.05	17	38.6	27	61.4	>0.05	2	4.5	42	95.5	>0.05
Have a partner	-	-	68	100.0		48	70.6	20	29.4		15	22.1	53	77.9		3	4.4	65	95.6	
**Professional Experience (years)**																				
1 to 15	2	2.8	70	97.2	>0.05	49	68.1	23	31.9	>0.05	20	27.8	52	72.2	>0.05	5	6.9	67	93.1	>0.05
16 or more	-	-	40	100.0		30	75.0	10	25.0		12	30.0	28	70.0		-	-	40	100.0	
**Weekly working hours**																				
< 40 h	-	-	11	100.0	>0.05	7	63.6	4	36.4	>0.05	4	36.4	7	63.6	>0.05	2	18.0	9	81.8	>0.05
40 h	2	2.0	99	98.0		72	71.3	29	28.7		28	27.7	73	72.3		3	3.0	98	97.0	
**Working shift**																				
Half time	-	-	7	100.0	>0.05	3	42.9	4	57.1	>0.05	1	14.3	6	85.7	>0.05	1	14.0	6	85.7	>0.05
Full time	2	1.9	103	98.1		76	72.4	29	27.6		31	29.5	74	70.5		4	3.8	101	96.2	

## Discussion

Analyzing the existence of occupational violence in the workplace is a difficult action, since it requires defining the concept of violence, as well as establishing a causal relationship between work and violence (17).

In the present study, the prevalence of occupational violence was 73.2%, similarly to that reported by other researchers (10,18). This suggests the need for greater attention to violence that occurs within the workplace. The prevalence of violence reported in other countries varied significantly, as observed rates of 27.7% in Egypt (19) and 76% in Hong Kong (9). However, the methodological differences between studies have made direct comparisons difficult. Additionally, differences within the reported prevalence of violence among different countries may be due to weekly workload, workplace organization and attitudes of victims in reporting the violence (15).

Although the reasons that led to elevated prevalence of occupation violence were not investigated in the present study, some hypothesis can be used to explain this condition: the current state of public services (including understaffing and inadequate work conditions); frequent shortages of medicines and supplies; overcrowded queues and delays in receiving care (20).

Verbal abuse was the most frequent form of abuse among the four types of workplace violence, which is consistent with previous studies (9,16,21). Similarly to an earlier report about the occupational violence in Thailand (15), physical injuries were also less recurrent. However, in the present study, the occupational violence was not statistically associated with the variables gender, professional experience, weekly working hours and working shift.

With regards to verbal abuse, typical perpetrators were patients and their relatives/caregivers (9,15,16,20). Some factors such as dissatisfaction with the type of service offered, the delay in treatment and the poor quality of health services in Brazil are hypotheses that may explain the aggressiveness of patients and their caregivers.

In contrast, psychological harassment was frequently practiced by heads of teams and another health professionals, which confirms the results of a previous Brazilian report (22). Once the psychological harassment is an existing type of violence in labor relations, the perpetrator may be the supervisor or co-worker himself (16). Among the possible factors that can lead to aggressions towards colleagues are stress and low job satisfaction (20).

This study showed that most verbal abuse and psychological harassment occurred during the morning shift, or during both morning and afternoon. This result is similar to study (8) that found primary care health services in Brazil work at both morning and afternoon shifts.

The main risk factors pointed by nurses at primary care were the lack of security and the violent behavior of patients and their caregivers. The most frequent origin of the abuse were patients (15), patient’s family (23), visitors (20) and other healthcare staffs. The effects of abuse on nurses produce the following conditions: exhaustion, sleeping disorders, nightmares, stress, continuous headaches, self-dissatisfaction, fear of work, depression, and others (8,24).

This study has limitations that affect the interpretation of results such as its cross-sectional design that limits cause and effect inferences, demonstrating exclusively the presence or absence of associations. In addition, the data collection method, use of self-report, may also be a limitation due to the memory biases, and this may have influenced our data on the prevalence of violence (20,21,25).

In Campina Grande, Brazil, the work of nurses at the primary care of public health could be affected by various occupational risk factors that cause health damage and interfere with the amount and quality of assistance provided to patients. Therefore, measures should be implemented to prevent the occurrence of violent acts, including the training of these workers to face critical situations; to improve working conditions and safety and creating a record protocol occurrences of ho occupational violence (26).

## Conclusion

The prevalence of occupational violence among Brazilian nurses is high, with predominance of verbal violence. Perpetrators are frequently the patients themselves and the lack of security within the workplace is the main reported risk factor.

## Ethical considerations

Ethical issues (Including plagiarism, informed consent, misconduct, data fabrication and/or falsification, double publication and/or submission, redundancy, etc.) have been completely observed by the authors.
